# Training gut and body: physical activity, diet, ASA and microbiota influence the outcome in gastrointestinal cancer survivors

**DOI:** 10.3389/fnut.2025.1648004

**Published:** 2025-10-16

**Authors:** Ester Oneda, Silvia Noventa, Michela Libertini, Sara Cherri, Alessandra Manno, Fausto Meriggi, Michele Martinetti, Alberto Zaniboni

**Affiliations:** ^1^Department of Clinical Oncology, Fondazione Poliambulanza, Brescia, Italy; ^2^Università Telematica San Raffaele, Rome, Italy

**Keywords:** physical activity, gastrointestinal cancer, gut microbiota, exercise in oncology, cancer survivorship, immunity, ASA, colorectal cancer

## Abstract

Gastrointestinal (GI) cancers remain a significant contributor to global cancer mortality. Recent evidence highlights the crucial role of lifestyle interventions—particularly physical activity and diet—in improving outcomes for GI cancer survivors. This comprehensive review explores how structured exercise, in combination with dietary strategies and selective pharmacologic interventions like aspirin, can modulate key biological processes including insulin sensitivity, inflammation, immune response, and gut microbiota composition. A central theme is the modulation of the gut microbiota. Physical activity and diet promote microbial diversity and the growth of species with anti-inflammatory and immunostimulatory properties—effects that may enhance therapeutic efficacy and resilience to treatment toxicity. The concept of a “trained microbiota,” inspired by studies in athletes, is proposed as a model to understand how lifestyle can durably shape host–microbe interactions. Furthermore, aspirin use in genetically selected populations shows promise in reducing recurrence, highlighting the potential for integrated, low-risk interventions. Finally, exercise improves quality of life, functional capacity, and treatment tolerance, while reducing fatigue and psychological distress. Translating these findings into practice requires structured integration into oncology care pathways, with multidisciplinary collaboration and tailored prescriptions of physical activity—combining aerobic and resistance training, nutritional support, and psychological care. Despite the need for further high-quality trials, especially in gastric and pancreatic cancer, current data provide a strong rationale for promoting lifestyle-based strategies as adjunctive therapy in gastrointestinal oncology. The review advocates for a paradigm shift in survivorship plans—one that integrates physical training, nutritional optimization, and microbiota support to enhance long-term outcomes in GI cancer survivors.

## 1 Introduction

Gastrointestinal (GI) cancers—including colorectal, gastric, esophageal, and pancreatic malignancies—are among the leading causes of cancer-related mortality worldwide. In recent years, there has been growing interest in lifestyle factors, such as physical activity and diet, in modulating prognosis following a GI cancer diagnosis. Numerous observational studies suggest that physically active cancer patients tend to have better survival outcomes compared to their sedentary counterparts (1–3).

Physical activity positively influences various physiological aspects that affect tumor growth, including insulin and glucose metabolism, immune function, inflammation, sex hormones, oxidative stress, genomic instability, and myokines (110).

There is strong and consistent evidence that physical activity reduces the risk of colon cancer by approximately 20–25% (4). This protective effect is observed in both occupational and recreational activities and may be greater in individuals who maintain consistent physical activity throughout their lives. Clinically, exercise can also mitigate treatment-related side effects, such as fatigue and sarcopenia, improving physical performance and nutritional status—factors that can influence a patient’s ability to tolerate postoperative adjuvant therapies.

Moreover, diet and exercise influence the gut microbiota—the ecosystem of microbes inhabiting our intestines—which, in turn, can interact with the immune system and potentially affect tumor progression. Beyond a lower incidence, groups with healthier lifestyles exhibit up to a 52% reduction in cancer mortality compared to those with less healthy lifestyles, with significant decreases in deaths from gastrointestinal cancers (5, 6).

This review examines the evidence on the effect of regular physical activity on survival in patients with gastrointestinal cancers, in particular on colon-rectal cancers, assessing whether the benefit is dose-dependent (i.e., greater with more exercise); the combined role of diet and exercise in favorably modulating insulin sensitivity, the tumor microenvironment, and the immune system, particularly through their impact on the gut microbiota; observations that these effects are particularly pronounced in athletes and the possibility of “training” the gut microbiota through regular physical activity. Unfortunately, evidence on pancreatic and esophageal cancers is scarce. Current studies mainly focus on multimodal rehabilitation programs aimed at reducing surgical complications and improving quality of life.

**Figure 1:**
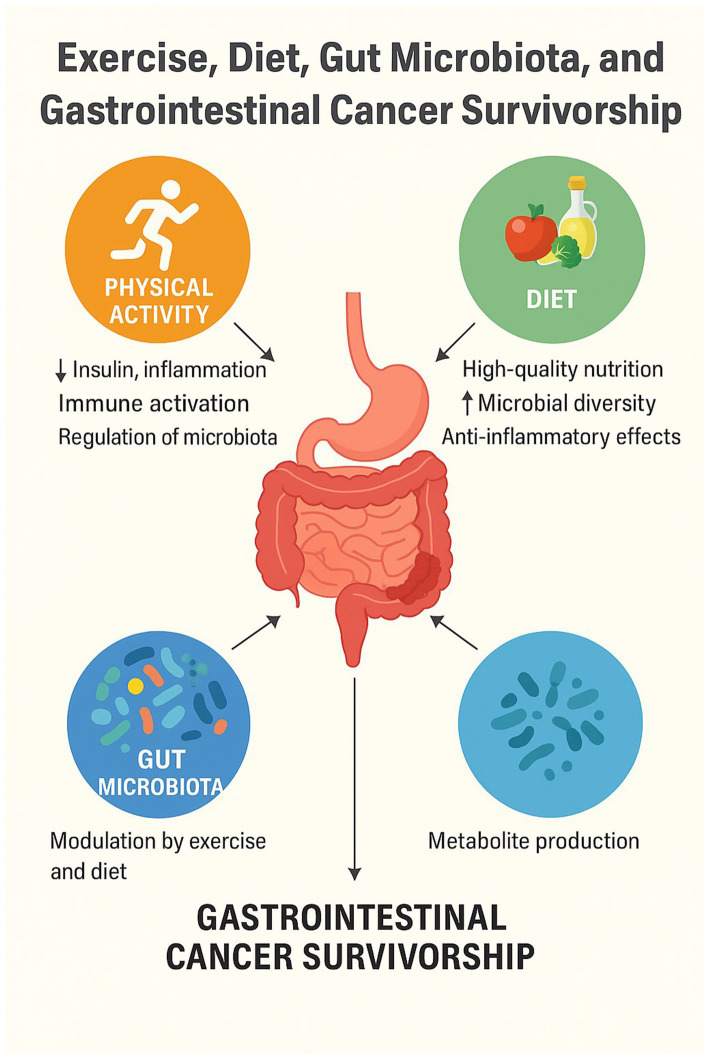
How exercise and diet impact GI cancer outcome.

This work summarizes recent scientific evidence on the role of physical exercise in preventing recurrences and improving survival and quality of life in patients with previous gastrointestinal tract neoplasms—diseases in which lifestyle is the most significant environmental predisposing factor. We will discuss the biological mechanisms underlying the observed benefits, results from clinical studies (RCTs and prospective studies) regarding disease-free survival (DFS), overall survival (OS), quality of life (QoL), and side effects, proposed exercise protocols, and strategies to integrate this evidence into clinical practice (Figure 1).

## 2 Physical activity and survival in gastrointestinal cancers

### 2.1 Colorectal cancer: benefits of exercise on survival

Colorectal cancer is the GI malignancy for which we have the most robust evidence regarding the benefits of physical activity on survival. A meta-analysis by Wu et al. (2) included over 17,000 patients with colorectal carcinoma and found that the most physically active individuals had approximately a 29% reduction in overall mortality compared to sedentary individuals (2). Specifically, regular exercise after a colorectal cancer diagnosis was associated with a 23% decrease in cancer-specific mortality risk (RR = 0.77) and a 29% decrease in all-cause mortality risk (RR = 0.71) compared to inactive patients (2). Similarly, physical activity practiced before diagnosis already showed a protective effect, with ~20% reductions in cancer-specific mortality among active individuals compared to sedentary ones (2). These data indicate that an active lifestyle, both before and after diagnosis, contributes to improved colorectal cancer prognosis.

Subsequent studies have confirmed and further quantified this benefit. An updated systematic analysis by Choy et al. (7) evaluated 13 prospective studies totaling over 19,000 patients who underwent curative surgery for colorectal carcinoma. The results clearly show improved postoperative survival associated with physical activity: patients engaging in moderate levels of physical activity had a hazard ratio (HR) for overall mortality of 0.82 (95% CI, 0.74–0.90) compared to those who were practically inactive, while patients with the highest levels of physical activity had an HR of 0.64 (95% CI, 0.56–0.72) (7). In other words, high physical activity after tumor resection was associated with an approximate 36% reduction in mortality, compared to an ~18% reduction in moderately active individuals, relative to sedentary patients (7). It’s important to note that the benefit of exercise was clearly evident in os and cancer-specific survival, while the effect on DFS was less consistent in this analysis (7). Conversely, a meta-analysis of prospective observational studies highlighted that post-diagnosis physical activity is associated with about a 30% reduction in colon cancer mortality risk and approximately a 38% reduction in all-cause mortality (8). Considering that specific mortality is closely related to the likelihood of recurrence, these data strongly support an improvement in DFS among physically active patients.

The CALGB 89803 trial has been one of the most significant studies in this area. In this study, involving patients who underwent surgery for stage III colon carcinoma treated with adjuvant chemotherapy, it was observed that those who engaged in regular physical activity had a significantly higher probability of remaining disease-free at 3 and 5 years compared to sedentary patients (9, 10). Similar observations have been reported in other cohorts: colorectal cancer survivors with high levels of physical activity have a 30–40% lower risk of recurrence compared to sedentary individuals (4). A systematic review published in 2025 analyzed the determinants of healthy lifestyles in a population of 2,134 colorectal cancer survivors, highlighting that at least 150 min per week of moderate physical activity reduce the risk of recurrence by 31%, through pathophysiological mechanisms such as regulation of insulin resistance and reduction of intestinal inflammation (11). Adherence to WCRF/AICR dietary recommendations, including higher intake of fruits, vegetables, and fiber and lower consumption of red and processed meat and alcohol, has been associated with a reduced overall cancer risk (HR 0.93; 95% CI 0.92–0.95) (12) and specifically a lower risk of colorectal cancer (HR 0.84–0.86; 95% CI 0.80–0.90) (13). This suggests that physical activity may contribute both to reducing recurrences and, more importantly, to improving the patient’s overall condition and ability to cope with the disease and treatments, translating into better long-term survival.

From a clinical perspective, this evidence has already influenced guidelines. Currently, cancer survivors are advised to avoid inactivity and engage in at least 150 min of moderate aerobic exercise per week (or ≥75 min of vigorous activity), in line with general population guidelines (7). These recommendations, issued by entities such as the American Cancer Society, stem from the observation that patients who achieve these minimum activity levels (e.g., brisk walking for ~30 min a day, 5 days/week) have significantly better outcomes. Interestingly, some studies suggest that post-diagnosis physical activity has an even greater impact on colorectal cancer prognosis than pre-diagnosis activity. In a comprehensive meta-analysis across various cancer types, the reduction in cancer-specific mortality risk associated with high levels of exercise after diagnosis was around 37% (HR ~ 0.63), more pronounced than the ~18% reduction associated with pre-diagnosis exercise (14). Specifically, in colorectal carcinoma, Friedenreich et al. (14) report that the HR for mortality was approximately 0.60 in patients who remained physically active after diagnosis, confirming the importance of intervention even after the disease onset (14).

Clinical studies like CALGB 89803 and CALGB 80702 have further explored these benefits. The combined analysis of 2,876 patients (mean age at diagnosis: 60.8 years; 56% men) showed that 44% engaged in 3–17.9 MET-hours/week of activity, 29.2% less than 3 MET-hours/week, and 26.8% at least 18 MET-hours/week. Patients with <3 MET-hours/week had a 3-year survival rate 17.1% (CALGB 89803) and 10.8% (CALGB 80702) lower than the general population, while this gap reduced to 3.5 and 4.4% in patients with ≥18 MET-hours/week. Among patients disease-free at 3 years, those maintaining ≥18 MET-hours/week even surpassed the general population’s survival (+2.9%) (15).

Supporting causality, *post hoc* analyses, such as one conducted on 1,696 patients operated for stage III colorectal cancer, showed that postoperative physical activity is associated with a significant reduction in recurrence risk, especially in the first year after surgery (HR ~ 0.68) (15). Recent high-quality evidence has reinforced the role of physical activity as a cornerstone of tertiary prevention in colorectal cancer survivors with an improvement of DFS and OS (16). These findings provide compelling level 1 evidence that structured exercise programs confer substantial oncologic and functional benefits and should be considered a critical component of survivorship care plans in patients with curatively treated colon cancer. Overall, for colorectal cancer, the evidence strongly supports the idea that “staying active” improves survival chances.

### 2.2 Other gastrointestinal cancers: evidence and limitations

While colorectal cancer is the most extensively studied, emerging research indicates benefits of physical activity in other gastrointestinal cancers, albeit with varying levels of evidence (Table 1). For gastric cancer, a recent meta-analysis by Ma et al. (17) demonstrated that physically active individuals have a lower risk of both developing the cancer and dying from it. Specifically, combined data suggest that high levels of physical activity are associated with approximately a 24% reduction in gastric cancer mortality risk (RR ~ 0.76) compared to low activity levels (Table 2) (17). This outcome partly stems from a lower incidence but also implies that among patients with gastric carcinoma, those who are more active may live longer. Some prospective studies have observed that gastric cancer patients who maintain light to moderate physical activity during therapies tolerate treatments better and exhibit a higher QoL which could indirectly translate into survival benefits.

**Table 1:** Strength of evidence for the impact of physical activity in GI cancers.

Cancer type	Survival outcome evidence	Quality of Life (QoL)	Biological plausibility (mechanisms)	Overall strength of evidence
Colorectal cancer	★★★★★ (Multiple RCTs & meta-analyses)	★★★★☆ (Fatigue, function, mood)	★★★★★ (Insulin/IGF axis, immunity, microbiota, perfusion)	Very Strong
Gastric cancer	★★☆☆☆ (Few cohort studies)	★★★★☆ (Post-gastrectomy rehab)	★★★☆☆ (Some preclinical data)	Moderate
Pancreatic cancer	★☆☆☆☆ (Limited, observational)	★★★☆☆ (QoL, fatigue)	★★★★☆ (Immunity, microbiota in preclinical studies)	Low–Moderate
Esophageal cancer	☆☆☆☆☆ (No survival trials)	★★★☆☆ (Rehab post-surgery)	★★☆☆☆ (Sparse, theoretical links)	Low
Hepatobiliary cancer	☆☆☆☆☆ (Sparse data)	★★☆☆☆ (Function, fatigue)	★★★☆☆ (Bile acid clearance, metabolism)	Low–Moderate

☆☆☆☆☆ = Very Weak | ★☆☆☆☆ = Weak | ★★☆☆☆ = Moderate | ★★★☆☆ = Strong | ★★★★☆ = Very Strong.

**Table 2:** Impact of physical exercise on the gut microbiota.

Effect of physical exercise on microbiota	Involved mechanism(s)
Increased alpha-diversity (73, 74)	Greater microbial ecosystem diversity
Increased SCFA-producing bacteria (acetate, propionate, butyrate) (6, 74)	Production of anti-inflammatory and barrier-protective metabolites
Reduced intestinal transit time	Less contact between intestinal mucosa and toxins
Increased intestinal blood flow and energy metabolism	Altered nutrient availability for intestinal microbes
Synergistic effect with fiber-rich diet (Mediterranean diet) (6)	Optimal substrates for microbiota-derived immune regulators
Antitumor immunity stimulation via IL-15/CD8 + axis (e.g., pancreatic carcinoma) (57, 114)	Adrenergic activation → IL-15 → cytotoxic T cells
Increased immunostimulatory bacteria (Akkermansia, Faecalibacterium, Fusibacteria) (58)	Microbiota profile shift toward immune-beneficial species
Potential improvement of chemotherapy tolerance (e.g., capecitabine) (61)	Increased microbial metabolic pathways (e.g., vitamin K₂ biosynthesis)
Systemic anti-inflammatory modulation (6, 73)	Microbial balance shift toward symbiotic species
Protective role against cancer and enhanced immune response (60)	Continuous microbiota-gut-immune system interaction

Regarding esophageal cancers, the evidence is more limited. It is known that regular physical exercise can help prevent gastroesophageal reflux and obesity, risk factors for esophageal adenocarcinoma. A meta-analysis indicated a potential protective effect of physical activity in reducing esophageal cancer risk (approximately 29% lower risk among the most active individuals) (18). However, in patients already diagnosed with esophageal carcinoma, there are no robust studies directly linking exercise to improved survival. Nevertheless, post-surgical rehabilitation interventions with home-based exercises have been shown to improve physical fitness and muscle function in esophageal cancer survivors, factors that could promote faster recovery and better capacity to undergo any adjuvant therapies (19). In summary, although research on the esophagus is still evolving, staying active appears beneficial for the patient’s overall health in this context as well.

Pancreatic carcinoma represents a particular case: it is often diagnosed at an advanced stage and has a severe prognosis, making lifestyle intervention studies challenging. Some epidemiological studies have suggested that regular physical activity can reduce the risk of developing pancreatic cancer by about 10%, especially in overweight individuals (20). In patients already affected, data are limited but indicate encouraging trends. Generally, exercise for pancreatic cancer patients is known to improve symptoms and QoL (e.g., reduced fatigue, improved muscle tone and appetite) more than having direct effects on the tumor itself. Interestingly, new preclinical studies are revealing immunological mechanisms through which exercise could positively influence even aggressive tumors like pancreatic cancer—a topic we will explore further later.

A crucial aspect in very advanced cancers is understanding how late initiating exercise can still offer an advantage. A systematic analysis by Takemura et al. (21) on patients with advanced/metastatic cancers (in various organs, including GI) highlighted mixed results: in their aggregated data, advanced-stage patients reporting higher levels of physical activity did show a trend toward lower mortality (combined HR ~ 0.78 for observational studies), but randomized clinical trials comparing exercise interventions to control did not demonstrate a statistically significant survival benefit (21). In other words, patients already in very advanced and debilitated stages may not derive a tangible life-prolonging benefit from starting an exercise program at that terminal phase (21), although they may still gain functional and symptomatic benefits. This underscores the importance of early initiation: integrating physical activity throughout the care pathway, ideally from the early stages (or even before diagnosis, as primary prevention), is likely necessary to fully reap survival advantages.

### 2.3 Dose-dependence of exercise benefits

A key question is whether the advantages offered by physical activity increase with the “dose” of exercise—that is, whether engaging in more activity leads to incremental survival benefits. Current evidence indeed suggests a dose–response relationship, though with potential plateau thresholds beyond certain levels. In the aforementioned study by Choy et al. (7) on colorectal carcinoma, it was observed that patients in the highest activity group had better outcomes than those in the moderate activity group, indicating that additional exercise conferred further benefits. A similar trend emerged from dose–response analyses in other contexts: for example, among breast and colon cancer survivors, Friedenreich et al. (14) found consistent reductions in mortality risk with increasing physical activity up to approximately 10–15 MET-hours/week, after which the benefit curve tended to stabilize. To provide a practical idea, 10–15 MET-hours/week roughly equate to 3–5 h of brisk walking (4 METs) per week, a volume close to standard recommendations. In other words, transitioning from a completely sedentary lifestyle to at least this amount of weekly exercise yields a marked survival gain; further increases (e.g., >20 MET-hours/week, corresponding to daily vigorous exercise) may offer additional benefits but with diminishing returns.

It’s important to emphasize that any amount of activity is better than none: many studies highlight that the most significant benefit jump occurs when comparing completely sedentary individuals to those engaging in even light daily movement. For instance, in colorectal carcinoma, moving from complete inactivity to a low-moderate activity level (e.g., a few hours of walking per week) is associated with significant mortality reductions (7). Subsequently, increasing from moderate to intense activity yields a further (but smaller) improvement. No deleterious effects of excessive exercise on cancer patients’ survival have been identified in the literature—provided that extreme or unsupervised exercise can pose injury or physical stress risks. In summary, the protective effect of physical activity in GI cancers appears dose-dependent: “some is good, more is better,” up to at least the guideline-equivalent levels of 150 min/week, beyond which benefits tend to saturate but do not disappear. This dose–response relationship provides further support for promoting exercise among cancer patients, encouraging them to progressively approach the upper tolerated limits of activity based on their clinical conditions.

## 3 Diet and physical activity: synergy and immunity headings

The beneficial effect of physical activity on survival in patients with gastrointestinal (GI) cancers may be partly mediated by lifestyle-induced biological changes such as reduced proliferative signaling, improved metabolic control, a less inflammatory environment, and enhanced immunity. These biological effects provide a solid rationale for the epidemiological observation of fewer recurrences in physically active patients.

### 3.1 Hormonal and metabolic modulation

Physical activity plays a crucial role in modulating insulin sensitivity, significantly influencing the prevention and progression of various types of cancer, particularly those of the gastrointestinal tract, such as colorectal cancer. Insulin and insulin-like growth factor 1 (IGF-1) are potent promoters of cell proliferation and tumor cell survival. Elevated circulating levels of these hormones, often found in conditions of insulin resistance and hyperinsulinemia, activate intracellular signaling pathways like PI3K/Akt/mTOR and RAS/MAPK, promoting tumor growth and inhibiting apoptosis (22, 23). Insulin itself functions as an oncogenic factor, facilitating neoplastic progression when present in excess. In experimental models, hyperinsulinemic states lead to constitutive activation of the Akt pathway with accelerated growth of colon tumors, while pharmacological inhibition of this pathway induces cell cycle arrest and apoptosis in neoplastic cells (24, 25). Similarly, the IGF-1-IGF1R binding activates pro-mitogenic signals overlapping those of insulin are correlated with a higher risk of developing tumors such as colorectal cancer (26).

Physical activity improves tissue sensitivity to insulin by 40–60%, helping to reduce both blood glucose levels and the pancreas’s insulin secretion needs (23). Trained muscles absorb more glucose in response to insulin and increase their oxidative capacity for free fatty acids. Regular exercise lowers basal levels of insulin and bioavailable IGF-1. Neoplastic cells receive fewer proliferative stimuli through their respective receptors, resulting in attenuated growth signals.

In mice genetically predisposed to developing intestinal polyps (Apc^Min/+ model), voluntary physical activity led to a marked favorable alterations in the insulin-hormonal profile (decrease in the serum IGF-1/IGFBP-3 ratio) and a significant reduction in the number of intestinal tumors compared to sedentary controls (27).

Changes in insulin and IGF-1 induced by physical activity are closely linked to body composition, as reductions in fat mass and gains in muscle mass improve metabolic health (28). These effects are more evident when exercise also promotes weight loss (29). In colorectal cancer survivors, aerobic programs of ~150 min/week have been shown to reduce fasting insulin (30, 31). The impact on the IGF-1 axis is more complex: while IGF-1 levels may increase, this is often accompanied by higher IGF-binding proteins, which can mitigate tumor-promoting effects (32). Overall, moderate-to-vigorous activity appears to exert the strongest influence on these hormonal pathways (31).

Higher circulating C-peptide and lower IGFBP-1 have been linked to poorer prognosis in colorectal cancer, supporting the role of hyperinsulinemia in tumor progression (33). Consistently, the survival benefit of exercise was most evident in tumors with low IRS-1 expression, suggesting reduced tumor sensitivity to insulin signaling (34). Clinical trials confirm these associations: in a phase II RCT, 6 months of moderate aerobic exercise reduced fasting insulin and HOMA-IR in colorectal cancer survivors, with greater benefits in those with higher exercise volumes (up to −20 pmol/L) (30). These findings indicate that physical activity can rebalance insulin metabolism even after diagnosis, contributing to improved outcomes. A 12-month exercise intervention reduced colonic crypt cell proliferation, providing direct evidence of the antiproliferative effect of exercise in humans (35). Other strategies targeting insulin resistance support this rationale: metformin has shown antitumor effects in preclinical models and improved outcomes in diabetic cancer patients (36, 37), whereas exogenous insulin use has been associated with a modestly increased risk of gastrointestinal tumors (38, 39). Similarly, bariatric surgery, which markedly lowers hyperinsulinemia, has been linked to reduced colorectal cancer incidence in obese individuals (40).

### 3.2 Inflammation and immunity

Moderate-intensity physical activity plays a significant anti-inflammatory role, contributing to the reduction of chronic inflammation markers such as interleukin-6 (IL-6), tumor necrosis factor-alpha (TNF-α), and C-reactive protein (CRP) (41). Concurrently, it enhances the efficiency of the immune system, improving surveillance mechanisms that can eliminate residual tumor cells or micrometastases, thereby reducing the risk of recurrence (42).

Physical activity induces the release of myokines such as IL-6, IL-15, and irisin (43, 44), which in preclinical studies show pro-apoptotic and anti-proliferative effects on tumor cells (45, 46). Regular exercise reduces CRP by 30–50% and increases anti-inflammatory cytokines like IL-10 (47, 48), while enhancing NK cell and CD8 + T cell activity (49, 50, 111) and modulating hematopoiesis toward an anti-inflammatory profile (51, 52). Collectively, these changes reduce proliferation and promote apoptosis in tumor tissues (53). Exercise also activates PPAR-γ, a key regulator of inflammation and colorectal carcinogenesis, particularly in obesity-related models (54). Clinically, a 16-week circuit training program lowered IL-6, IL-8, TNF-α, and hsCRP in obese, sedentary cancer patients (55), consistent with meta-analyses showing exercise effectively reduces systemic inflammatory markers (56).

#### 3.2.1 Role of the microbiota

Beyond direct effects, physical activity beneficially modulates the gut microbiota, leading to the production of microbial metabolites with anti-inflammatory properties that may protect against tumor development and progression.

The gut microbiota is a complex ecosystem of trillions of microorganisms (bacteria, viruses, fungi) residing in our intestines, contributing to vital functions such as digestion, nutrient metabolism, inflammation modulation, and immune system development. Environmental factors like diet are known modulators of the microbiota: for instance, a fiber-rich diet promotes bacteria that produce short-chain fatty acids (SCFAs) like butyrate, while a Western diet high in fats and red meats can encourage pro-inflammatory microbial populations.

Less intuitively, but increasingly documented, is that physical exercise also influences the composition and activity of the gut microbiota. Numerous studies, both in animal models and humans, indicate that regular exercise increases microbial diversity and the presence of beneficial bacteria in the gut. For example, physical activity promotes the growth of bacteria that produce anti-inflammatory metabolites such as SCFAs (acetate, propionate, and butyrate), which help maintain the integrity of the intestinal barrier and positively modulate the local immune response.

Moreover, exercise can reduce intestinal transit time, decreasing the contact duration of potential toxins with the mucosa and creating an internal environment favorable to a balanced microbiota. Additionally, engaging in physical activity enhances intestinal blood flow and energy metabolism, factors that can alter nutrient availability for microbes and thus indirectly reshape the microbial ecosystem.

These effects will be analyzed in subsequent sections.

The greatest benefits on the gut microbiota occur when exercise is combined with a healthy diet. A review by Zhang et al. (6) showed that this synergy more effectively improves microbial composition, host metabolism, and inflammatory status. Diets rich in anti-inflammatory compounds, such as the Mediterranean diet, provide substrates for beneficial microbes, while exercise promotes their use to generate metabolites like butyrate, which support immune regulation. Together, nutrition and exercise can “train” the microbiota toward a balanced state, enhancing local and systemic immunity (6). Greater microbial diversity correlates with improved antitumor surveillance, as commensal species stimulate cytokine release and dendritic cell activation. Exercise further strengthens immunity indirectly by shaping the microbiota and directly by boosting antioxidant and immune function.

Recent studies highlight the microbiota’s role in modulating therapy response. Certain bacterial taxa are linked to better efficacy of immune checkpoint inhibitors in melanoma and other cancers, and similar research is underway in gastrointestinal tumors. In pancreatic cancer models, aerobic exercise increased CD8 + T cell infiltration via adrenaline-mediated activation of the IL-15 axis, enhancing responsiveness to immunotherapy (6, 57). Parallel findings show that exercise enriches anti-inflammatory and immunostimulatory species such as *Akkermansia muciniphila*, *Fusibacteria*, and *Faecalibacterium prausnitzii*, associated with more effective immune profiles against cancer (58).

High dietary sugar intake has been linked to more aggressive early-onset colorectal cancer (EOCRC): patients with high-sugar diets showed a higher rate of metastatic disease at diagnosis (90.1% vs. 79.1%) (59). Excess sugar may promote systemic inflammation, creating a pro-tumorigenic environment. EOCRC also displays distinct microbiome and immune profiles compared with late-onset CRC, including enrichment of pro-inflammatory taxa, depletion of regulatory phyla (e.g., Firmicutes, Bacteroidetes), reduced alpha diversity, and increased inflammatory gene expression. These features were associated with aggressive, immune-depleted subtypes (CMS2/3) (60), suggesting that dysbiotic microbiota may contribute to EOCRC pathogenesis.

Microbiome–treatment interactions further highlight its clinical relevance. Capecitabine therapy has been shown to shift microbial taxa and enhance pathways for menaquinone (vitamin K₂) biosynthesis, which correlated with reduced chemotherapy-related toxicities, particularly peripheral sensory neuropathy (61). These adaptations may improve treatment tolerance and outcomes. In this context, physical activity could further support microbiome diversity and colonization by beneficial taxa, potentially augmenting chemotherapy efficacy and reducing toxicity.

Therefore, while diet and exercise are well established as beneficial for overall health, emerging evidence also suggests that they may jointly modulate the gut microbiota in ways that could enhance immune function. This potential interaction could contribute to reduced tumor recurrence and improved treatment outcomes.

### 3.3 Vascular adaptations of the tumor microenvironment

Regular physical activity has been shown to positively modulate the tumor microenvironment (TME), particularly by enhancing tumor vascularization, improving blood perfusion, and reducing intratumoral hypoxia. Chronic exercise increases tumor microvessel density, promoting the formation of more functional blood vessels within tumors, thereby improving oxygen and nutrient delivery (62).

Furthermore, exercise enhances intratumoral blood flow and decreases hypoxic regions, which are often associated with more aggressive tumor behavior and increased resistance to cancer therapies (62, 63). Regular physical activity also supports the development of more mature and functional blood vessels, characterized by reduced vascular resistance and improved overall conductance (63, 64).

These vascular changes contribute to optimizing the delivery of oxygen and nutrients to tumor tissues, potentially making neoplasms more susceptible to anticancer treatments (62, 63). Studies in murine models have demonstrated a synergistic effect between physical activity and chemotherapy, leading to prolonged disease progression times (62).

Specifically, moderate-intensity exercise has proven effective in preventing tumor dissemination by normalizing angiogenesis and reducing endothelial cell permeability, thereby limiting potential metastasis formation (65).

Finally, physical activity stimulates the secretion of myokines and microRNAs from skeletal muscle, molecules with anti-oncogenic properties capable of activating antitumor immune responses, further contributing to the favorable modulation of the tumor microenvironment (66).

### 3.4 Enhancement of hepatobiliary function

Animal studies have demonstrated that chronic voluntary physical activity significantly increases the biliary excretion of endogenous bile acids. Rats subjected to exercise exhibited notably higher bile acid excretion compared to sedentary controls, indicating an enhancement of hepatobiliary function in response to physical activity (67, 68). Specifically, an increase in basal bile flow and improved clearance of bile acids such as taurocholate were observed, suggesting an augmented capacity of the liver to process and eliminate bile acids. Additionally, exercise enhanced the elimination of other substances, including glutathione and certain organic anions, reflecting an overall improvement in hepatobiliary efficiency (67, 68).

In the context of colon cancer, physical activity may positively influence bile acid metabolism and intestinal transit. These effects can reduce the duration of exposure of the intestinal mucosa to potentially carcinogenic compounds, including secondary bile acids. Such mechanisms, while particularly relevant in the preventive phase, may also play a role in preventing recurrences or the formation of new lesions following an initial neoplasm (69).

## 4 Gut microbiota and sports: the case of athletes and the “gut training”

A unique opportunity to observe the long-term and extreme effects of exercise and diet on the gut microbiota is to study elite athletes. These individuals, particularly those engaged in endurance sports, combine high volumes of training with controlled nutritional regimens, offering a model of how lifestyle can shape the microbiota. The concept of a “trained” gut microbiota arises from observations that athletes exhibit a distinctly different and potentially healthier microbial profile compared to sedentary individuals.

A pioneering study by Clarke et al. (70) compared the gut microbiota of professional rugby players with that of non-athlete controls. The findings revealed that athletes possessed greater microbial diversity, with 22 distinct bacterial phyla identified, compared to 11 or fewer in sedentary controls. This enriched diversity is considered a marker of ecological robustness, as a more diverse microbiota can perform a broader range of metabolic functions and offers greater resilience against stressors such as suboptimal diets or diseases. Furthermore, the study noted that in rugby players, the abundance of certain “beneficial” bacteria positively correlated with protein intake and blood levels of creatine kinase (a marker of muscle load), suggesting that both the high-protein diets typical of athletes and intense training contribute to shaping their microbial ecosystem. Notably, athletes showed increased presence of species from the genera Akkermansia and Faecalibacterium, known for their anti-inflammatory properties and support of intestinal barrier integrity (70).

Additional research has confirmed that the “athlete’s microbiota” possesses unique characteristics: higher levels of butyrate-producing bacteria, a balanced Firmicutes to Bacteroidetes ratio, and a greater abundance of species involved in the metabolism of branched-chain amino acids and other metabolites beneficial to performance (71). A study by Grosicki et al. (72) even identified a specific microbe that may confer advantages to endurance athletes. By analyzing marathon runners, the researchers discovered an increased proportion of Veillonella in their intestines post-race. Veillonella is capable of metabolizing lactic acid produced during exercise. When a strain of *Veillonella atypica* isolated from athletes was administered to laboratory mice, the rodents exhibited a significant improvement in running endurance. Metabolic analysis revealed that Veillonella utilized lactate as a carbon source, converting it into propionate, a short-chain fatty acid. Direct administration of propionate into the colons of mice mimicked the endurance-enhancing effect, suggesting a beneficial cycle: intense exercise produces lactate, which nourishes Veillonella in the colon; Veillonella proliferates and produces propionate; propionate, in turn, enhances exercise efficiency by potentially boosting muscle capacity or reducing inflammation (72).

The notion of a “trained microbiota” implies that regular physical activity induces lasting adaptations in the gut microbiota, similar to how it trains the heart, muscles, and lungs. These adaptations include increased diversity, a rise in beneficial species, a reduction in potentially harmful ones, improved metabolic efficiency (e.g., in processing metabolites like lactate), and even enhanced production of host-beneficial molecules (such as propionate in the aforementioned example). Importantly, these effects are not exclusive to elite athletes. Studies on previously sedentary individuals who commence exercise programs have observed positive changes in their microbiota within weeks. A 2023 systematic review (73) found that moderate-to-high intensity exercise protocols, performed three or more times per week for at least 8 weeks, can significantly alter the gut microbiota in both healthy adults and clinical populations. These changes often include increased alpha-diversity (within-sample diversity) and shifts in the abundance of key bacterial genera. Interestingly, the benefits to the microbiota appear largely reversible: if exercise is discontinued, the microbiota tends to revert to its original configuration over time (74). This reinforces the analogy with physical training: just as athletic fitness requires consistency, maintaining a “fit” microbiota necessitates the continuation of healthy lifestyle habits.

In conclusion, studies in athletes illustrate the potential of diet and exercise to shape the gut microbiota, but these findings represent an extreme model. Importantly, similar benefits may also be achievable in the general population through gradual adoption of regular physical activity and balanced dietary patterns. In oncology, it is neither realistic nor advisable for patients to emulate athletic regimens; rather, the underlying principles—improved fitness, adequate nutrition, and greater microbial diversity—can be adapted in a tailored manner. Such approaches may contribute to reduced inflammation, enhanced immune competence, and better resilience during treatment. However, these hypotheses remain largely supported by observational or preclinical evidence, and robust randomized controlled trials in cancer patients are still needed before firm conclusions on clinical outcomes can be drawn.

### 4.1 Improvement of quality of life (QoL)

Numerous randomized controlled trials (RCTs) have demonstrated that physical exercise programs, initiated during or after oncological treatments, significantly enhance the quality of life (QoL) of patients with gastrointestinal tumors, including colorectal cancer. The benefits manifest both physically (increased energy, functional recovery, ability to perform daily activities) and psychologically (reduction of anxiety and depression). A meta-analysis confirmed that regular aerobic and/or resistance exercises lead to significant improvements in QoL scores and physical functionality compared to controls (75).

#### 4.1.1 Reduction of cancer-related fatigue

Cancer-related fatigue is one of the most common and debilitating side effects following chemo-radiotherapy, especially in gastrointestinal tumors. Physical exercise is currently the most effective non-pharmacological intervention to combat fatigue. Studies on patients with colorectal carcinoma in follow-up have confirmed a decrease in chronic fatigue and an increase in energy in subjects assigned to regular physical activity compared to sedentary individuals (76–78). These positive effects are also observed in colorectal cancer survivors: postoperative training programs have shown better perceptions of general health status and fewer persistent symptoms, such as pain or digestive issues.

#### 4.1.2 Specific benefits on bone health, muscle mass, and cardiometabolic function

Weight-bearing exercises improve bone density and reduce the risk of therapy-induced osteopenia/osteoporosis. Additionally, physical exercise helps maintain or increase muscle mass, counteracting sarcopenia and cancer cachexia, particularly relevant in advanced pancreatic tumors (79, 80). Aerobic programs can mitigate the negative cardiovascular effects of certain therapies, contributing to blood pressure, lipid, and glycemic control, and enhancing the overall wellbeing of survivors.

#### 4.1.3 Improvement of functional capacity and autonomy

Regular physical exercise improves functional capacity and the maintenance of autonomy in daily activities, helping patients better tolerate treatments and maintain independence (81–84). Furthermore, exercise can reduce cancer-related symptoms such as fatigue, neuropathy, and constipation, further supporting QoL (82, 83, 85).

#### 4.1.4 Improvement of sleep and psychological wellbeing

Data indicate that regular physical activity improves sleep quality and reduces insomnia in cancer patients, as well as decreasing stress and symptoms of anxiety and depression. Both standard exercise and mind–body approaches (such as Qigong) are effective in reducing sleep disturbances, with standard exercise providing greater improvements in perceived strength and exercise capacity (84, 86, 87). Better sleep contributes to recovery and can positively influence immune and hormonal mechanisms.

#### 4.1.5 Reduction of other symptoms

Some toxicities, such as chemotherapy-induced peripheral neuropathy (e.g., oxaliplatin in colorectal cancer patients), currently lack clear evidence of benefits from exercise; studies so far are inconclusive or insufficient (88). Similarly, for effects like nausea, chronic pain, cognitive dysfunction (“chemobrain”), or sexual dysfunctions, data do not allow us to affirm that exercise produces significant improvement. However, the absence of definitive evidence does not imply evidence of no effect: these areas remain open to research.

#### 4.1.6 Specific benefits for gastrointestinal cancer subtypes

**Colorectal Cancer:** Pelvic floor rehabilitation (PFR) has been shown to alleviate symptoms of Low Anterior Resection Syndrome (LARS), a common postoperative complication in rectal cancer patients. A systematic review by Chan et al. (89) concluded that PFR can significantly improve bowel function and quality of life in these patients.

**Gastric Cancer:** Postoperative exercise programs have demonstrated benefits in patients recovering from gastrectomy (90, 112). For instance, Cho et al. (91) implemented a structured exercise regimen, including in-hospital and home-based exercises, for gastric cancer patients undergoing minimally invasive gastrectomy. The study found that participants experienced improved physical fitness, muscle strength, and quality of life without any adverse events.

**Pancreatic Cancer:** Integrating nutritional support with physical exercise has shown promise in managing cachexia and improving quality of life in pancreatic cancer patients. Weyhe et al. (92) conducted a randomized controlled trial where patients undergoing pancreatic resection participated in an intensive physiotherapy program. Results indicated significant improvements in physical functioning, appetite, and overall quality of life.

These findings underscore the importance of incorporating tailored exercise and nutritional programs into the care plans for gastrointestinal cancer patients to enhance recovery and overall wellbeing.

## 5 Integrated exercise protocols in gastrointestinal oncology

Over recent years, accumulating evidence has led to the development of international multidisciplinary guidelines advocating for structured physical activity among cancer survivors. These guidelines recommend that, health permitting, oncology patients engage in at least 150 min of moderate-intensity aerobic activity (e.g., brisk walking) per week, or 75–150 min of vigorous-intensity aerobic activity, complemented by muscle-strengthening exercises on at least 2 days per week. Additionally, balance training (particularly for older adults to prevent falls) and flexibility exercises are advised (Table 3).

**Table 3:** Training programs in CRC.

Protocol type	Frequency/duration	Main benefits
Supervised aerobic + resistance (78)	2×/week, 18 weeks	↓ Fatigue, ↑ physical function, cost-effective
Resistance training (93)	2×/week, 3–5 sets, moderate-heavy	Maintains muscle, supports chemo completion
Multimodal (endurance, resistance, balance) (85)	2×/week, 8 weeks	↓ Neuropathy, ↑ balance/strength
Home-based/low-intensity (15, 94)	Varies	Maintains fitness, safe

### 5.1 Multimodal prehabilitation and rehabilitation in gastrointestinal cancer surgery

For patients scheduled for gastrointestinal cancer surgery, multimodal prehabilitation programs aim to optimize physical and psychological readiness both before and after the surgical intervention. These programs typically encompass:

- **Physical Exercise:** High-intensity aerobic and resistance training performed 2–3 times per week, either under hospital supervision or through structured home-based sessions
- **Nutritional Support:** Individualized dietary counseling and supplementation, often emphasizing high protein intake (e.g., daily whey protein), to support muscle mass maintenance and recovery.
- **Psychological Support:** Interventions aimed at reducing anxiety, enhancing coping strategies, and, when necessary, supporting smoking cessation to improve mental wellbeing and stress management.

### 5.2 Timing and delivery modalities

- **Prehabilitation (Preoperative Phase):** Implementing programs 2–4 weeks prior to surgery has been associated with significant improvements in functional capacity and reductions in major postoperative complications compared to standard care or postoperative-only rehabilitation.
- **Rehabilitation (Postoperative Phase):** While beneficial, initiating multimodal interventions solely after surgery tends to be less effective in enhancing walking capacity and functional recovery than prehabilitation.
- **Supervision:** Supervised sessions, whether in clinical settings or through professional oversight, generally yield better adherence and outcomes. However, home-based programs with regular monitoring are also effective, particularly for previously inactive patients.

### 5.3 Clinical evidence from randomized controlled trials

#### 5.3.1 Exercise programs

Colon cancer patients are encouraged to follow supervised exercise programs, especially those combining aerobic and resistance training, which are most effective at reducing fatigue, improving physical function, and managing chemotherapy side effects. These training programs are evaluated in RCTs:

- Supervised Combined Aerobic and Resistance Training: A 18-week program, twice weekly, 1-h sessions that includes both aerobic and resistance exercises significantly reduces physical and general fatigue, and improves physical functioning during and after chemotherapy (78). This strategy is cost-effective and feasible for colon cancer patients.
- Resistance Training: Moderate to heavy loads (65–85% of one-repetition maximum): 3–5 sets of 6–10 repetitions, twice weekly. It focus on large, multijoint movements (e.g., squats, presses) and can be supervised or home-based. The adherence is supported by trainer visits or remote guidance (93). This training aims to maintain muscle mass, prevent dose-limiting toxicities, and support chemotherapy completion (93).
- Multimodal Exercise (Endurance, Resistance, Balance): 8-week supervised program, 2×/week, 60 min per session. It includes endurance, resistance, and balance training and helps stabilize or improve chemotherapy-induced peripheral neuropathy, balance, and strength (85).
- Home-Based and Low-Intensity Programs: Home-based or low-intensity supervised programs are also safe and feasible. These programs can help maintain or improve fitness and manage fatigue, though effects may be smaller than supervised, higher-intensity protocols (15, 94).

The CHALLENGE trial, a multicenter phase III randomized study conducted in 889 patients with resected stage III or high-risk stage II colon cancer, evaluated a three-year structured aerobic and resistance exercise program initiated shortly after adjuvant chemotherapy. Across 55 international centers, the intervention significantly improved DFS compared to health education alone (5-year DFS: 80.3% vs. 73.9%; HR 0.72, 95% CI 0.55–0.94), and 8-year OS (90.3% vs. 83.2%; HR 0.63, 95% CI 0.43–0.94). Participants also achieved clinically meaningful gains in cardiorespiratory fitness, physical functioning, and activity levels, while adverse musculoskeletal events were infrequent and manageable. The exercise protocol combined progressive aerobic training (up to 300 min/week) with bi-weekly strength sessions, beginning under supervision and transitioning to a maintenance phase supported by behavioral coaching. Adherence was high—79% for supervised sessions and 83% for behavioral support in the first 6 months—and remained substantial over 3 years (44 and 63%, respectively). Notably, sustained engagement in moderate-to-vigorous activity correlated with greater physiological improvements, which in turn translated into superior oncologic outcomes. These findings provide level 1 evidence that structured, behaviorally supported exercise should be integrated into standard survivorship care for colon cancer patients (16). While the trial was not designed to assess a formal dose–response effect, the findings suggest that both the intensity and durability of physical activity influence clinical benefit. These results emphasize the critical importance of sustained behavioral support and individualized exercise planning to optimize adherence and maximize oncologic outcomes.

Other integrated protocols adopt a multimodal approach: “prehabilitation” before major surgery—including respiratory and aerobic exercises to improve preoperative cardiopulmonary capacity—followed by postoperative rehabilitation involving progressively intensified exercises (57, 95, 96). This integrated strategy has demonstrated improved functional recovery and may hypothetically translate into fewer complications and long-term recurrences.

In pancreatic cancer survivors, low-intensity programs have been proposed due to the high prevalence of frailty. These include resistance exercises using elastic bands or light weights to preserve muscle mass, short daily walks to counteract deconditioning, and yoga or tai chi to improve balance and reduce stress. Although large trials are lacking, these personalized protocols have shown feasibility and improvements in parameters such as strength and exercise tolerance among participating patients.

Prehabilitation programs have been shown to reduce serious postoperative complications, enhance physical performance (e.g., walking distance), and improve quality of life (97–101). The addition of supervised exercises may be particularly beneficial for sedentary patients (96, 102). However, no clear benefit has been demonstrated regarding a further reduction in hospital length of stay or mortality, and effects in frail elderly patients remain uncertain (101, 103, 104).

Personalizing programs based on individual patient factors (e.g., age, comorbidities, cancer stage, ongoing side effects) is crucial. Engaging physiotherapists or certified cancer exercise specialists to assess patients and develop safe training plans is recommended. Generally, combining aerobic and strength training yields the most comprehensive benefits across cardiovascular, muscular, and metabolic domains. Group sessions can offer motivation and social support, while home-based exercises with telemonitoring enhance practicality. Many protocols adopt a hybrid model, starting with supervised sessions in clinical settings, followed by home-based maintenance, demonstrating good adherence and outcomes.

Safety assessments are integral to integrated protocols. Over the past decade, data have consistently shown that exercise is generally safe for cancer survivors, including during treatment, when appropriate precautions are taken. Protocols typically include exclusion criteria (e.g., severe anemia, acute infections, uncontrolled heart failure) and guidelines for adjusting intensity in response to symptoms (e.g., excessive fatigue, pain). No randomized controlled trials have reported significant adverse events attributable to exercise in intervention groups compared to controls, reinforcing the notion that moderate physical activity is a safe adjunct to standard cancer care.

#### 5.3.2 Aspirin and diet: uninflammatory action

In the current era of precision oncology, it is increasingly evident that complementary strategies to standard treatment are essential to improve both survival and quality of life in patients with gastrointestinal (GI) cancers. The recently published ALASCCA trial provides robust evidence for one such approach: daily use of low-dose aspirin (160 mg) for 3 years significantly reduced colorectal cancer recurrence in patients with tumors harboring PI3K pathway mutations. In this multicenter, biomarker-driven phase 3 study involving 626 patients with stage I–III rectal or stage II–III colon cancer across Scandinavia, aspirin lowered the 3-year recurrence rate by 51% in patients with PIK3CA exon 9/20 mutations and by 58% in those with other PI3K alterations, reducing recurrence to 7.7% in both groups (105). Importantly, the benefit was consistent across subgroups regardless of disease stage, tumor location, or adjuvant therapy. These findings underscore the importance of upfront genomic profiling and the potential of low-risk, repurposed agents in cancer prevention and survivorship. In summary, anti-inflammatory, antiplatelet and immunomodulatory properties of aspirin could be beneficial in PIK3CA-mutant tumors due to their reliance on inflammatory and PI3K-driven pathways. ESMO and ASCO guidelines suggest potential benefit in secondary prevention for select populations but no routine recommendation. Reasonably, low-to-intermediate doses (75–160 mg/day) could be suggested for chemoprevention, especially in patients with cardiovascular indications or molecular predictors (e.g., PIK3CA mutation). Risk–benefit assessment is crucial before prescription, especially in older patients or those with bleeding risks.

Complementing these pharmacologic strategies, recent observational data highlight the significant prognostic impact of lifestyle-based anti-inflammatory interventions. Malnutrition and muscle mass loss are consistently associated with reduced treatment tolerance, higher complication rates, impaired quality of life, and shorter survival (2, 7, 15). Targeted dietary interventions—such as ensuring a protein intake of 1.5–2 g/kg/day from varied sources—have proven effective in limiting sarcopenia, sustaining immune function, and improving tolerance to chemotherapy and radiotherapy (9, 10, 16). Adopting a predominantly plant-based dietary pattern, particularly the Mediterranean diet, has been associated with better body composition, reduced systemic inflammation, improved quality of life, and lower all-cause mortality among gastrointestinal cancer survivors (5, 6, 12, 13). Conversely, consumption of red and processed meats, ultra-processed foods, and alcohol has been linked to increased recurrence risk and cancer-related mortality, and is therefore strongly discouraged according to international recommendations (4, 11). A varied diet rich in fruits, vegetables, whole grains, legumes, and fish also contributes to gut microbiota modulation and chronic inflammation reduction, ultimately supporting both prognosis and overall wellbeing in cancer patients (1, 14).

In a prospective cohort analysis of 1,625 patients with stage III colon cancer enrolled in the CALGB/SWOG 80702 trial, individuals consuming highly pro-inflammatory diets—characterized by frequent intake of red and processed meats, refined grains, and sugar-sweetened beverages—had an 87% higher risk of death compared to those adhering to anti-inflammatory diets rich in dark leafy greens, yellow vegetables, coffee, and tea. Notably, the best survival outcomes were observed among patients who combined a low-inflammatory diet with regular physical activity (≥9 MET-hours/week), resulting in a 63% reduction in mortality risk relative to sedentary patients with inflammatory diets (106). Although no significant difference in disease-free survival was observed, the improvement in overall survival points to the broad systemic effects of chronic inflammation and its modulation through behavior. Although, in this study daily use of celecoxib and low-dose aspirin (<100 mg/d) did not affect the association between inflammatory diet and survival (106).

Together, these findings suggest that an integrated care model—combining molecularly guided pharmacologic prevention (e.g., aspirin), anti-inflammatory nutrition, and structured physical activity—could form a potent, non-toxic triad to enhance prognosis in GI cancer survivors. This multifaceted approach, tailored to both tumor biology and patient lifestyle, represents a paradigm shift in survivorship care aimed not only at reducing recurrence but also at optimizing long-term health and wellbeing.

## 6 Discussion: integration of evidence into clinical practice and limits

The recognition of physical activity as a critical component of cancer care has led to the development of guidelines by various international oncology organizations. In 2018, the Clinical Oncology Society of Australia (COSA) issued a position statement advocating for the prescription of exercise to all cancer patients as an integral part of their treatment regimen. This stance is supported by numerous health institutions and underscores the importance of incorporating exercise into standard cancer care practices.

Similarly, the American College of Sports Medicine and the American Cancer Society have published guidelines emphasizing the role of clinicians in encouraging and facilitating physical activity among cancer patients. These guidelines collectively promote the view that exercise should be considered a “prescribed” adjunct therapy in oncology, tailored to individual patient needs and conditions.

To effectively integrate exercise into cancer care, organizational changes are necessary. Proposed models of “rehabilitative oncology” involve collaboration among specialists such as physiatrists, kinesiologists, and physiotherapists with oncology training, working alongside oncologists and surgeons. Practical steps include:

- **Screening and Counseling During Visits**: Assessing patients’ activity levels during follow-up visits using questionnaires and providing personalized counseling to safely increase physical activity.
- **Structured Exercise Programs**: Establishing dedicated exercise programs within hospitals or community settings, including supervised sessions post-therapy. Some centers have introduced specialized gyms or partnerships with fitness facilities for cancer patients. Participation in walking groups, gentle exercise classes, or yoga tailored for oncology patients are additional implemented strategies.
- **Multidisciplinary Collaboration**: Involving nutritionists to support increased energy needs, psychologists to enhance motivation and address psychological barriers to exercise, and sports medicine physicians to monitor physiological adaptations. This holistic approach maximizes the benefits of exercise within the overall care plan.

Effective integration also necessitates training healthcare professionals. Historically, physical activity received limited attention in oncology; however, recent guidelines and consensus statements, such as the 2019 international consensus on exercise in cancer survivors, urge clinicians to “prescribe movement” akin to pharmacological treatments. Initiatives like workshops, continuing medical education courses, and practical manuals assist oncologists in acquiring basic competencies to provide tailored exercise recommendations.

Despite robust evidence that exercise improves outcomes, implementing exercise programs for gastrointestinal (GI) cancer patients in outpatient settings is challenging due to multiple real-world barriers. Many patients have significant comorbidities and treatment-related side effects that limit their exercise capacity (107). Adherence to prescribed exercise regimens is often suboptimal; fatigue and other chemotherapy toxicities frequently lead to low participation or drop-out (113). Socioeconomic and geographic disparities further impede access—patients from rural or low-resource areas who lack nearby facilities or transportation tend to be less active. Motivational barriers are also prevalent, as patients may have low interest, limited self-efficacy, or fear that exercise could worsen their symptoms without proper guidance. At the health system level, inadequate infrastructure and a shortage of specialized staff (such as physiotherapists or exercise physiologists trained in oncology) hinder routine integration of exercise medicine into oncology care.

To overcome these barriers, various evidence-based strategies have been proposed and tested. Individually tailored exercise prescriptions that account for a patient’s comorbid conditions, treatment side effects, and baseline fitness are recommended to enhance safety and engagement (108). For example, a recent trial in metastatic cancer patients found that allowing individuals to choose their preferred exercise format (home-based vs. supervised training) resulted in high attendance (median ~92%) and adherence (~88%), with corresponding improvements in fitness and QoL (109). Telehealth models are increasingly used to deliver exercise programs remotely, which can bypass geographic/logistical barriers and have shown comparable efficacy in improving physical fitness while increasing accessibility for those unable to attend in-person sessions. In addition, behavioral support interventions such as health coaching and tele-coaching are being explored to sustain long-term exercise adherence; although one six-month remote coaching program did not significantly boost post-rehabilitation activity levels, this approach highlights the ongoing efforts to address motivation and habit formation. Multidisciplinary interventions that embed exercise specialists within oncology clinics and provide coordinated support (including caregiver encouragement) have been identified as key facilitators of patient participation. Overall, several barriers to implementation exist: limited dedicated resources, variability in patients’ physical conditions, and psychological resistance (e.g., fear of injury, lack of exercise habits). Current randomized trials and systematic reviews indicate that with tailored programming, telehealth delivery, behavioral coaching, and better institutional support, many of the traditional barriers to exercise in GI cancer populations can be mitigated, enabling more patients to safely reap the benefits of physical activity during and after treatment. Implementation science studies suggest initiating pilot programs, actively involving patients—potentially through peer coaching from former patients sharing positive experiences—and incorporating physical activity into care plans at the point of discharge following active treatments.

By adopting these strategies, healthcare systems can effectively integrate exercise into cancer care, enhancing patient outcomes and quality of life.

## 7 Conclusion

**Figure 2:**
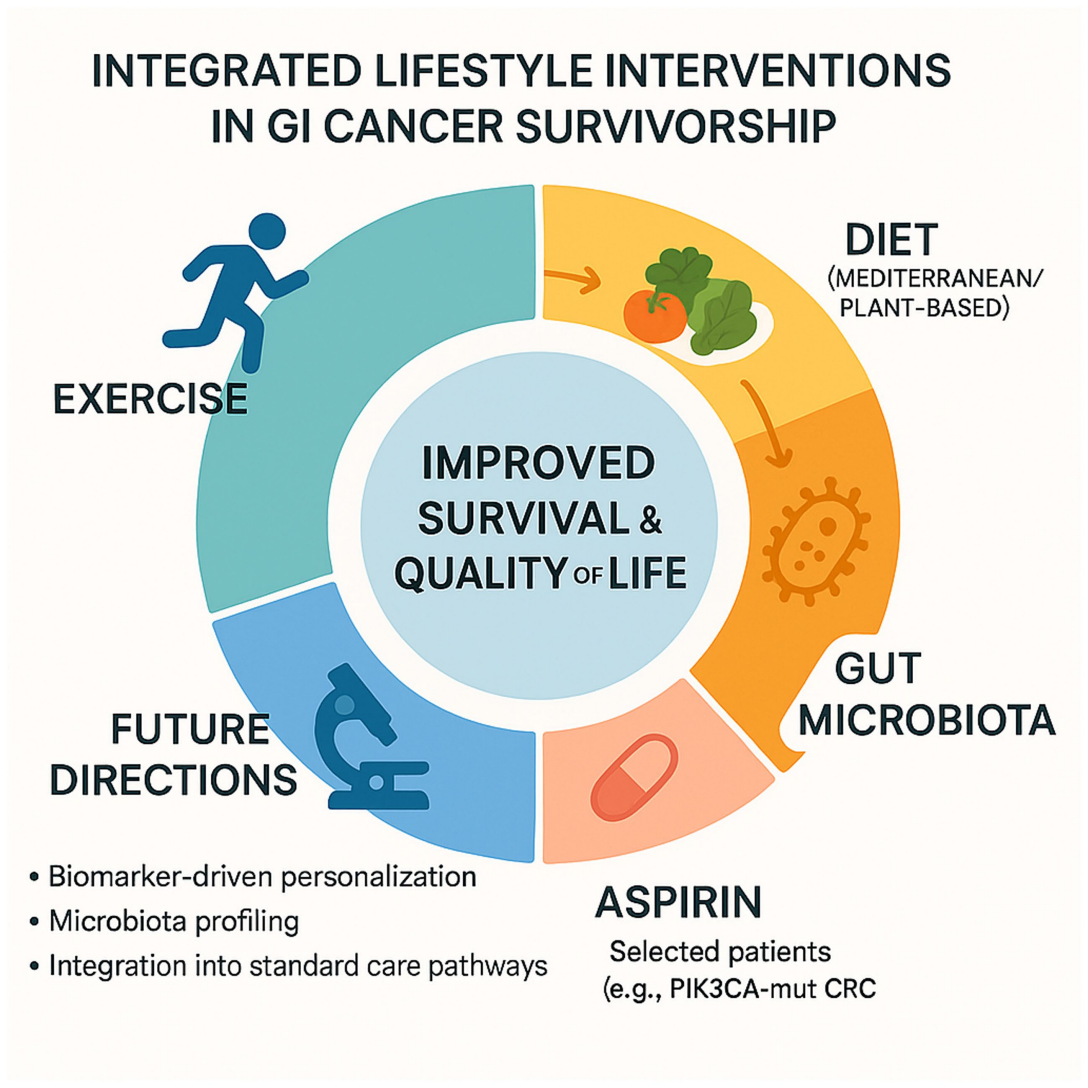
Integrated lifestyle interventions in GI cancer survivorship.

In summary, the growing body of clinical and translational evidence strongly supports the role of lifestyle interventions as an essential component of survivorship care in gastrointestinal cancers. Physical activity, diet, and adjunctive strategies such as low-dose aspirin act through converging biological pathways—including modulation of insulin sensitivity, reduction of systemic inflammation, and reshaping of the gut microbiota—contributing to improved survival, quality of life, and treatment tolerance (Figure 2).

Building upon current clinical and translational evidence, several key takeaways can guide the integration of lifestyle interventions into the care of gastrointestinal cancer patients:

- Strong evidence supports the integration of structured exercise programs in colorectal cancer (CRC) survivorship. Randomized controlled trials such as the CHALLENGE study demonstrate improved disease-free survival, quality of life, and treatment tolerance, particularly when combining aerobic and resistance training.
- Moderate evidence exists for gastric cancer, with emerging studies suggesting potential benefits of physical activity and nutritional strategies, though further randomized trials are needed to validate long-term oncologic outcomes.
- Limited but promising data are emerging for pancreatic and esophageal cancers, particularly in the context of localized disease amenable to neoadjuvant chemotherapy. In these settings, structured prehabilitation protocols—combining physical, nutritional, and psychological optimization before surgery—have shown early signs of improving treatment tolerance and enhancing functional recovery, warranting further exploration in controlled trials.
- An integrated model—combining physical activity, dietary modulation (e.g., Mediterranean diet), and low-dose aspirin in selected patients—emerges as a promising strategy to shape the gut microbiota, reduce inflammation, and enhance anti-tumor immune surveillance, especially in biomarker-defined populations (e.g., PIK3CA-mutant CRC).
- Future directions include biomarker-driven personalization of lifestyle interventions, gut microbiota profiling, and incorporation of exercise and diet into standard oncologic care pathways. Clinical trials such as ADD-ASPIRIN and translational studies on the concept of a “trained microbiota” will inform next-generation survivorship strategies.

Together, these insights call for a paradigm shift in oncology care: moving beyond a sole focus on pharmacologic therapies to a truly integrated model in which structured exercise, nutritional optimization, and selective pharmacological adjuncts are embedded within survivorship plans. Such a holistic approach has the potential to not only extend survival but also to improve the quality and resilience of life in gastrointestinal cancer patients.
